# Experiences in Awake Craniotomy from Borneo: A Case Series from Sarawak General Hospital

**DOI:** 10.21315/mjms2024.31.5.16

**Published:** 2024-10-08

**Authors:** Kugan Vijian, Bik Liang Lau, Davendran Kanesen, Swee San Lim, Peter Chee Seong Tan, Donald San Liew Ngian, Albert Hieng Sii Wong

**Affiliations:** 1 Department of Neurosurgery, Sarawak General Hospital, Sarawak Malaysia; 2 Department of Anaesthesiology, Sarawak General Hospital, Sarawak Malaysia

**Keywords:** awake craniotomy, Borneo, case series

## Abstract

**Background:**

The indications for awake craniotomy now spans from resection of tumours at eloquent areas of the brain, deep brain stimulation and treatment of aneurysms to name a few. In the region of East Malaysia where patients have various ethnic backgrounds and native languages, planning and execution of these procedures can be somewhat challenging.

**Methods:**

This is a retrospective analysis of 11 awake surgeries conducted by the Department of Neurosurgery in Sarawak. The indications for awake craniotomy surgery in our sample population were intra-axial lesions in eloquent regions involving important cortical areas and subcortical tracts which were at risk of damage during tumour excision. Patients were assessed for intra-operative and post-operative neurological deficits.

**Results:**

Eleven patients aged 20 years old–70 years old were included in this series. All patients were diagnosed with lesions in eloquent areas of the brain requiring surgical excision. Patients were of various ethnic backgrounds. The spoken language of these patients also varied based on their ethnicity. The histopathological diagnosis of nine patients were consistent with gliomas with three being of high grade. Three patients (27%) developed intra-operative deficits that were not present pre-operatively.

**Conclusion:**

This case series serve to demonstrate the feasibility of awake craniotomies even in centres without vast experiences in awake surgeries and ideal adjuncts which in comparison may be readily available in different centres. Although careful patient selection has been emphasised, it is a difficult feat in a region consisting of at least 30 different ethnic groups with distinct languages and cultures.

## Introduction

Horsley (1) pioneered the awake craniotomy technique, publishing a series of 10 cases in 1887. The detailed methodology and safety aspects of these procedures were elaborated on in his publication. Since then, this new surgical technique, which was initially used to treat epilepsy (2), has gained considerable popularity. Currently, the indications for awake craniotomy include resection of tumours in eloquent areas of the brain, deep brain stimulation and aneurysm clipping (3, 4). Its deserving fame is attributed to several important advantages, including the real-time assessment of patients’ motor and speech functions, and decreased post-operative recovery time (3). Evidently, these intra-operative assessments require advanced medical equipment and imaging which may include electrophysiological monitoring and cortical mapping, as well as tractography, which are essential for preserving important functions and preventing new post-operative deficits.

The outcomes of awake craniotomies are highly dependent on careful patient selection and thorough pre-operative planning (5). The patient selection criteria primarily encompass the ability of the patient to tolerate surgery in an awake state, without a breach or difficulty in communication between the doctor and patient during surgery. Communication barriers were assessed and evaluated pre-operatively. Spoken language, which is the most common and important form of communication, may differ according to dialect and vocabulary, which can interfere with the transmission and reception of information during surgery. Other patient characteristics, such as anxiety and impaired cognition, would, in essence, exclude them from awake surgery. Detailed patient counselling and discussions with the anaesthetic team are necessary to ensure a smooth intra-operative and post-operative course. From an aesthetic standpoint, standard anaesthetic care involves a scalp block in combination with monitored anaesthesia care (MAC) or general anaesthesia (5–7).

In Malaysia, awake craniotomies have been performed in a few neurosurgical units with good results (8). Planning and execution of these procedures can be challenging in East Malaysia, where patients are of various ethnic backgrounds with their own distinct languages. Owing to this diversity in culture and language, patient selection plays a pivotal role in ensuring the success of surgery.

## Methods

This study comprised a retrospective analysis of all awake craniotomy surgeries conducted at the Neurosurgery Department of Sarawak General Hospital. This case series included 11 patients treated in our hospital between 2017 and 2022. Sarawak General Hospital is the largest tertiary hospital in the state of Sarawak. All patients who underwent awake craniotomies for various indications were included in this study.

### Patient Selection

The patients included in this study were diagnosed radiologically with intra-axial tumours or vascular lesions in the eloquent areas. These lesions showed no significant dural involvement and were not radiologically deemed highly vascular. Patients were all older than 18 years old and underwent surgery in the supine position.

Exclusion criteria included patients with anxiety or cognitive impairment, severe language barriers, overweight or obese patients with difficult airways, or significant comorbidities, large tumours or tumours deemed highly vascular on imaging, and extra-axial tumours.

### Pre-Operative Assessment and Counselling

All patients underwent assessment at our neurosurgical clinic, where they were assessed for suitability and subsequently counselled to undergo awake surgery. Patients were briefed on the importance and concepts of awake surgery and evaluated for their ability to follow verbal instructions correctly and promptly. They were also seen by an anaesthetist and explained in detail the anaesthesia and sedation aspects of the surgery. For patients with primary languages other than Bahasa Melayu, English or Mandarin, a designated translator was assigned to improve the intra-operative communication.

All patients underwent magnetic resonance imaging (MRI) of the brain with different proportions of patients aided with diffusion tensor imaging (DTI) and functional MRI (fMRI). The intra-operative neurophysiological monitoring (IONM) methods included motor evoked potential (MEP), somatosensory evoked potential (SSEP), free-running electromyography (EMG), cortical and subcortical mapping via direct electrical stimulation (DES), and central sulcus mapping with the aid of subdural grids.

During tumour resection, the patients were constantly engaged and assessed for any new neurological deficits pertaining to the cortical area of interest. In the event of a new weakness or speech deficits observed intra-operatively, surgical resection of the related area was not continued. The area of resection was also limited if a cortical or subcortical region achieved a motor threshold (MT) upon stimulation or if it comprised important white matter tracts on intra-operative tractography. Post-operatively, patients were assessed for new neurological deficits and residual tumours using post-operative MRI. The excised lesions were subjected to histopathological examination by pathologists and patients were subsequently referred to an oncologist when necessary. All procedures were performed using the awake-awake-awake method with dexmedetomidine infusion combined with targeted controlled infusions (TCI) of propofol and remifentanil, with the depth of sedation assessed using the bi-spectral index.

### Outcomes

Patients were assessed for residual tumours and new neurological deficits via post-operative imaging.

The collected data included sociodemographic details, clinical presentation, lesion characteristics, operative and anaesthesia notes, and post-operative notes. The data were tabulated and analysed using Microsoft Excel.

## Results

Table 1 presents the patient characteristics. Eleven patients aged 20 years old–70 years old were included in this study. All patients were diagnosed with lesions in the eloquent areas of the brain that required surgical excision. Nine (82%) were male. The patients came from various ethnic backgrounds, including Malay, Chinese, Iban, Lun Bawang and Orang Ulu, with Chinese and Iban accounting for the largest proportions, with four and three patients, respectively. The spoken language of the patients also varied according to their ethnicity. Most patients (55%) initially presented with seizures.

As shown in Table 2, the lesions were most commonly located in the motor and pre-motor areas (81%) and more than half (55%) of these lesions were smaller than 10 cm^3^, with the largest tumour volume being approximately 38 cm^3^ (Table 3). All patients underwent the awake-awake-awake technique in addition to a scalp block. However, one patient (Patient 4) was converted to general anaesthesia (GA) because of intra-operative brain swelling.

The patient converted to GA during awake surgery was a 69 year-old Chinese gentleman who presented with left-sided body weakness for a duration of 1 month. Imaging revealed a lesion in the right motor area. During pre-operative counselling for awake surgery, the patient was noted to have pre-morbid anxiety and barriers to communication. However, we proceeded with awake surgery as these issues were not severe. Intra-operatively, the patient was calm during the initial awake stage with monitored sedation. Access was achieved via a U-shaped skin incision and frontoparietal craniotomy. Subsequently, at the start of the resection, the patient failed to follow the instructions and became restless. We observed swelling of the brain without any evidence of clinical seizures, arterial or venous injury, or respiratory distress. Hence, the patient was eventually intubated and placed under GA to complete the resection of the tumour. Tumour resection was completed uneventfully under GA.

The mean duration of surgery was 218 min, with five cases lasting for more than 4 h. DTI was employed in eight patients and cortical/subcortical mapping in three of the 11 patients.

As shown in Table 4, three patients (27%) developed intra-operative deficits that were not present pre-operatively. The histopathological diagnoses of nine patients were consistent with gliomas, three of which were high-grade (WHO Grades 3 and 4). Seven patients are currently undergoing follow-up for surveillance to detect recurrence or an increase in the residual tumour. Table 5 shows an analysis of the factors which may have contributed to post-operative residual tumours or new neurological deficits.

## Discussion

This case series demonstrates the feasibility of awake craniotomies even in centres without extensive experience in awake surgeries and ideal adjuncts which, may be comparatively more readily available in different centres. Although careful patient selection has been emphasised by many authors, it is difficult to achieve at our centre as we cater to a region comprising at least 30 different ethnic groups with distinct languages and cultures (9). As shown in Table 1, the patients’ spoken languages varied from Bahasa Melayu to different ethnic mother tongues, such as Iban, Lun Bawang and Kayan. Although most patients had a fair command of Bahasa Melayu (the national language), we made a particular point to provide translators for each patient with mother tongues of languages other than which we were familiar with to minimise any form of break in communication or misinterpretation. It is worth noting that all the translators were medical staff. Several published studies, particularly those from Iran and Pakistan, have addressed these issues by providing translators and excluding patients who do not speak their respective national languages (10, 11). Patient comfort during surgery is given priority as it directly contributes to the tolerance and outcome of the procedure (12). In regards to this, all patients were given a brief layout of the operation theatre and the sequence of procedures that would be performed. The staff involved in surgery were also encouraged to introduce themselves to the patient pre-operatively to bridge any form of unfamiliarity or insecurity. Unnecessary conversations and sounds were also minimised as numerous studies have demonstrated that patients are significantly affected and disturbed by them (13, 14).

A trained and experienced anaesthetic team is vital to the success of awake craniotomies, as intra-operative complications and pain control need to be adequately addressed and dealt with (11, 15). Patients in our case series were anaesthetised and monitored by a neuroanathetist. As shown in Table 2, the technique utilised in our patients involved the monitoring of anaesthesia care with the addition of a scalp block. Anxious patients and the unfamiliarity of junior team members with awake craniotomies are among the challenges encountered. Steps which were taken to overcome these hurdles were the selection of the right candidates collectively performed by the neurosurgeons and anaesthesiologist, repeated pre-operative visits personally paid by the anaesthesiologist in charge to build a good rapport, as well as to ensure adequate psychological preparation. Perioperative debriefings are mandatory. Anaesthetic and surgical plans and the possibility of peri-operative complications were discussed before the start of anaesthesia and surgery, with a clear allocation of roles and responsibilities. Debriefing was also encouraged at the end of surgery to address any space for improvement. All patients were highly satisfied with the procedure.

The primary goals of awake surgery are to preserve important functions and prevent new post-operative debilitating deficits. This has become increasingly important in cases involving the cortical and subcortical areas that serve motor and speech functions. Neurological deficits can be caused either by the destruction of these areas or by oedema, with the latter usually being a temporary cause. One prior from India demonstrated that 23.8% of a sample population suffered from non-permanent neurological deficits, while another study reported a lower incidence of 8.5% (16, 17). These differences have been attributed to variations in techniques and sample sizes (16). In our series, three patients developed new deficits post-operatively, manifesting as motor weakness and dysphagia. In one of these patients, in whom IONM with cortical/subcortical mapping was utilised, the deficits subsequently resolved during follow-up. This is likely due to the ability to limit resection to areas devoid of positive EMG or MEP readings. Prior research has further reported significant benefits of motor mapping, such as a decrease in the post-operative complication rate from 21% to 13% when adequate neuromonitoring is present (18). Subdural grids and direct electrical stimulation were used to map the motor cortex and the corticospinal tract. During subcortical lesion resection with the aid of a monopolar probe around important white matter tracts, resection was halted once an MT of 7 milliampere (mA), which corresponds to a distance of 7 mm from the stimulated tracts. Extending the resection beyond this limit has been associated with mostly permanent motor deficits. Electrophysiological recordings and cortical stimulation, along with intra-operative assessments by neurophysiologists are performed in most established neurosurgical centres (8). The emergence of studies demonstrating added benefits and better outcomes with intra-operative functional-MRI (19) has also gained attention as more research is focused on areas of intra-operative safety and precise localisation.

## Conclusion

Awake craniotomies are becoming increasingly popular in the armamentarium of neurosurgeons because of their proven benefits. While advancements in medical technology play a prominent role in performing these procedures, our centre in Sarawak has also conducted them using the equipment, neuro-navigation and neurophysiological monitoring systems available to us, as is evident in this case series.

## Figures and Tables

**Table 1:** Patient characteristics (*N* = 11)

Variable	Frequency (%)
Age (mean)	44.5
Gender	
Male	9 (82)
Female	2 (18)
Ethnicity	
Malay	2 (18)
Chinese	4 (36)
Iban	3 (27)
Lun Bawang	1 (9)
Orang Ulu	1 (9)
Spoken language	
Bahasa Melayu	2 (18)
Mandarin	4 (36)
Bahasa Iban	3 (27)
Bahasa Kanyan	1 (9)
Bahasa Lun Bawang	1 (9)
Education level	
Primary school	4 (36)
Secondary school	4 (36)
Tertiary education	3 (27)
Initial presentation	
Incidental	1 (9)
Seizures	6 (55)
Weakness	3 (27)
Speech disturbances	1 (9)

**Table 2:** Tumour and perioperative characteristics (*N* = 11)

Variable	Frequency (%)
Tumour location	
Motor area	4 (36)
Pre-motor area	5 (45)
Speech area	2 (18)
Tumour size	
> 10 cm^3^	5 (45)
< 10 cm^3^	6 (55)
Adjuncts	
Cortical/Subcortical mapping	3 (27)
DTI/fMRI	8 (73)
Anaesthesia	
Awake-awake-awake	11 (100)
Conversion to GA*	1 (9)
Scalp block	11 (100)
Intra-operative deficits	
Paresis	2 (18)
Dysphasia	1 (9)
Duration of surgery	
Mean (min)	218
> 240 min	5 (45)
Post-operative deficits	
Paresis	3 (27)
Dysphasia	1 (9)
Post-operative duration of hospital stay (mean)	6.3 days
Histopathology	
Low grade glioma (diffuse astrocytoma, oligodendroglioma)	6 (55)
High grade glioma (anaplastic astrocytoma, glioblastoma)	3 (27)
Metastatic tumour	1 (9)
Cavernoma	1 (9)
Residual tumour (post-op scan)	4 (36)

Note:

* Patient 4, converted to GA due to intra-operative brain swelling (description in text)

**Table 3:** Summary of patient and tumour characteristics

Patient	Age	Gender	Ethnic	Presentation	Symptom duration	Tumour location	Tumour size (cm)Approximate vol. (cm^3^)	DTI/fMRI/IONM
1	35	Male	Iban	Incidental	N/A	Left premotor area	2.0 × 2.0 × 2.0 (4)	nil
2	37	Male	Lun Bawang	Seizure	4 months	Left premotor area	4.0 × 4.0 × 3.8 (30)	nil
3	20	Male	Orang Ulu	Seizure	1 year	Left superior temporal gyrus	4.7 × 3.7 × 2.8 (24)	nil
4	69	Male	Chinese	Left sided weakness	1 month	Right motor area	1.0 × 1.1 × 1.1 (0.5)	DTI
5	49	Male	Malay	Seizure	1 week	Right premotor area	5.8 × 3.2 × 4.0 (37)	DTI
6	45	Male	Chinese	Speech disturbance	6 months	Right superior temporal gyrus (right lobe dominant)	3.2 × 3.2 × 3.2 (16)	fMRI/ DTI
7	29	Male	Chinese	Seizure	9 months	Left motor area	4.8 × 3.9 × 4.1 (38)	fMRI/ DTI/ cortical-subcortical mapping
8	48	Female	Iban	Right sided weakness	1 month	Left motor area	2.4 × 2.4 × 2.6 (7)	DTI/ cortical-subcortical mapping
9	44	Female	Iban	Seizure	9 years	Right premotor area	1.5 × 2.0 × 2.0 (3)	DTI
10	70	Male	Malay	Left sided weakness	1 week	Right premotor area	2.5 × 2.1 × 2.4 (6)	DTI
11	44	Male	Chinese	Seizure	8 months	Right motor area	3.0 × 1.5 × 3.7 (8)	fMRI/DTI/cortical-subcortical mapping

**Table 4:** Peri-operative summary

Patient	Duration of surgery (minutes)	Intra-op deficits or complication	Post-op deficits	Post-op stay	HPE	Residual tumour	Progress and outcome
1	170	nil	nil	5 days	Cavernous angioma	No	Well and asymptomatic
2	205	nil	Right upper limb monoparesis	13 days	Oligodendroglioma WHO Grade 2	Yes	On surveillance-persistent right upper limb weakness
3	130	nil	nil	7 days	Diffuse astrocyctoma WHO Grade 2	No	On surveillance
4	190	Brain swelling	nil	8 days	Anaplastic astrocytoma WHO Grade 3	Yes	Debulking under GA for recurrence. Passed away after 1 year
5	250	nil	nil	3 days	Diffuse astrocytoma WHO Grade 2	No	On surveillance-seizure free
6	150	Left sided body weakness and dysphasia	Left upper limb monoparesis and dysphasia	5 days	Oligodendroglioma WHO Grade 2	No	On surveillance-resolved weakness and speech deficit
7	265	Right finger weakness	Right finger flexion weakness	2 days	Diffuse astrocytoma WHO Grade 2	No	On surveillance-resolved weakness
8	160	nil	nil	10 days	Metastatic adenocarcinoma	No	Underwent WBRT
9	330	nil	nil	3 days	Oligodendroglioma WHO Grade 2	No	On surveillance-Seizure free
10	280	nil	nil	6 days	Glioblastoma WHO Grade 4	Yes	Refused chemotherapy and radiotherapy
11	270	nil	nil	7 days	High grade glioma WHO Grade 3	Yes	Completed chemotherapy and radiotherapy-on surveillance

**Table 5:** Comparisons of patients’, tumour characteristics and outcomes

Variable	Tumour residual, frequency (%)	

	Yes	No
Lesion size		
> 30 cc	1 (25)	3 (43)
< 30 cc	3 (75)	4 (57)
Lesion location		
Motor area	2 (50)	2 (29)
Non-motor area	2 (50)	5 (71)
Use of brain mapping		
Yes	1 (25)	2 (29)
No	3 (75)	5 (71)

	**Presence of new post-operative deficits, frequency (%)**	

	**Yes**	**No**

Presence of pre-op deficits		
Yes	1 (33)	2 (25)
No	2 (67)	6 (75)
Lesion size		
> 30 cc	2 (67)	2 (25)
< 30 cc	1 (33)	6 (75)
Lesion location		
Motor area	1 (33)	3 (38)
Non-motor area	2 (67)	5 (62)
Use of brain mapping		
Yes	1 (33)	2 (25)
No	2 (67)	6 (75)
